# ^18^F–THK–5351, Fluorodeoxyglucose, and Florbetaben PET Images in Atypical Alzheimer’s Disease: A Pictorial Insight into Disease Pathophysiology

**DOI:** 10.3390/brainsci11040465

**Published:** 2021-04-06

**Authors:** Sohee Park, Minyoung Oh, Jae Seung Kim, Jae-Hong Lee, Young Wook Yoon, Jee-Hoon Roh

**Affiliations:** 1 Department of Neurology, Asan Medical Center, University of Ulsan College of Medicine, Seoul 05505, Korea; nrsoheep@naver.com (S.P.); jhlee@amc.seoul.kr (J.-H.L.); 2 Department of Nuclear Medicine, Asan Medical Center, University of Ulsan College of Medicine, Seoul 05505, Korea; my@amc.seoul.kr (M.O.); jaeskim@amc.seoul.kr (J.S.K.); 3 Department of Physiology, Neuroscience Institute, Korea University College of Medicine and School of Medicine, Seoul 02841, Korea; ywyoon@korea.ac.kr

**Keywords:** ^18^F–THK–5351 PET, atypical Alzheimer’s disease (AD), posterior cortical atrophy (PCA), logopenic variant of primary progressive aphasia (lpvPPA)

## Abstract

The recent advance of positron emission tomography (PET) tracers as biomarkers in Alzheimer’s disease (AD) provides more insight into pathophysiology, preclinical diagnosis, and further therapeutic strategies. However, synergistic processes or interactions between amyloid and tau deposits are still poorly understood. To better understand their relationship in focal brain changes with clinical phenotypes, we focused on region-specific or atypical AD characterized by focal clinical presentations: Posterior cortical atrophy (PCA) and logopenic variant of primary progressive aphasia (lpvPPA). We compared three different PET images with ^18^F–THK–5351 (tau), ^18^F–Florbetaben (amyloid beta, Aβ), and ^18^F–Fluorodeoxyglucose (glucose metabolism) to investigate potential interactions among pathologies and clinical findings. Whereas the amyloid accumulations were widespread throughout the neocortex, tau retentions and glucose hypometabolism showed focal changes corresponding to the clinical features. The distinctly localized patterns were more prominent in tau PET imaging. These findings suggest that tau pathology correlates more closely to the clinical symptoms and the neurodegenerative processes than Aβ pathology in AD.

## 1. Introduction

The main neuropathological hallmarks of Alzheimer’s disease (AD) are amyloid beta (Aβ) senile plaques and neurofibrillary tangles (NFT) [1,2]. To assess these pathologies noninvasively and preclinically, several amyloid and tau tracers have been developed recently which have enabled the visualization of regional distribution of amyloid or tau depositions in vivo [3,4]. However, the relationship between amyloid and tau in AD is still unknown and defining the relationship between the two will enable better understanding of the pathophysiology of the disease [1,5].

Aβ was thought to precede the accumulation of tau pathology followed by clinical symptoms, including memory impairment [1,4]. On the other hand, age-associated accumulation of NFT in normal elderly individuals has also been described and was introduced in the field as “primary age-related tauopathy (PART)” [6]. Now, it is conceived that the aggregation of Aβ triggers profound aggregation of NFT over the status of PART and further propagation of tau pathology in the brain, which corresponds well with clinical findings [1]. However, the spatial disparity in the accumulation of Aβ and NFT pathology in typical AD patients suggests that there are additional factors triggering the accumulation of each pathology and the interaction of the two. In this report, we attempted to focus on region-specific or atypical AD brain changes and investigated whether local interactions exist among Aβ, tau pathology, and brain functions in focal AD syndromes.

We investigated two focal cortical AD syndromes, posterior cortical atrophy (PCA) and logopenic variant of primary progressive aphasia (lpvPPA), which mainly share AD pathology with distinct clinical presentations [7,8]. Compared to the memory predominant presentations in typical late onset AD, the focal or atypical AD syndromes have distinctive phenotypes. PCA presents with visuospatial dysfunction and Gerstmann syndrome due to damages in the posterior cortical regions [7], whereas lpvPPA presents with word-finding deficits with prominent repetition errors related to the impairment in language-associated brain areas [8].

Here, we report two cases each for PCA and lpvPPA to compare the distinct imaging patterns using ^18^F–Florbetaben (FBB, Aβ), THK–5351 (tau), and Fluorodeoxyglucose (FDG, glucose metabolism) positron emission tomography (PET) images. We aimed to investigate whether the regional accumulation of Aβ overlaps with tau accumulation or glucose hypometabolism in focal AD syndromes. Then, we sought to determine whether the distribution of tau accumulation and glucose hypometabolism would have more regional association with clinical symptoms than amyloid PET uptakes.

Although the THK–5351 tracer was introduced in the field as one of the first-generation tau PET tracers [9], it was found to capture monoamine oxidase-B (MAO-B) instead of binding specifically to NFT [10]. However, the potential of the THK–5351 tracer to reflect astrogliosis in addition to tau pathology is now considered to be a promising imaging marker for detecting neuroinflammation in the brain [11]. In this study, we interpreted the ^18^F–THK–5351 uptakes in the brain as tauopathy, which includes but is not limited to actual regions of NFT accumulation.

## 2. Materials and Methods

### 2.1. Subjects

The participants were recruited from the Florbetaben Imaging in Alzheimer’s and Related Neurological Conditions (FLORIAN) cohorts at Asan Medical Center, Seoul, Korea [12]. All patients underwent precise history taking, neurologic examination, detailed neuropsychological tests, structural brain magnetic resonance imaging (MRI), and 3 different PET scans. The neuropsychological test was taken by the Seoul Neuropsychological Screening Battery (SNSB) [13]. This quantitative test measures attention, verbal and visual memory, frontal-executive function, visuospatial function, language, emotion, and general cognitive function, including Mini-Mental State Examination and the Clinical Dementia Rating. 

The study was conducted according to the guidelines of the Declaration of Helsinki and was approved by the Institutional Review Board of Asan Medical Center, Seoul, Korea. Informed written consent was obtained from all subjects involved in the study.

### 2.2. MRI Acquisition

Each patient underwent high-resolution T1, T2-weighted structural MRI scans using a 3T Philips Achieva MRI scanner with an 8-channel head coil (Achieva Release 1.0; Philips Medical Systems, Best, The Netherlands). A high-resolution anatomical 3-dimensional (3D) volume image was obtained using a 3D gradient-echo T1-weighted sequence. The following parameters were used to obtain images: Repetition time/echo time, 9.9/4.6 ms; flip angle, 8°; field of view, 224 × 224 mm; matrix, 224 × 224; and slice thickness, 1 mm with no gap. 

### 2.3. ^18^F–THK–5351 PET Imaging Acquisition

The ^18^F–THK–5351 PET imaging was performed on participants using the Discovery 690, 710, or 690 Elite PET/CT scanners (GE Healthcare) at Asan Medical Center. The PET images were obtained for 20 min, starting 50 min after injection of 185 ± 18.5 MBq of ^18^F–THK–5351. The final PET image was formed by summation of all the individual images. The quality of representative images was reviewed by 2 professional nuclear medicine board-certified physicians (M.O. and J.S.K.) for motion, image contrast, and noise. The PET images of all the participant were determined to be qualitatively adequate. 

### 2.4. ^18^F–Fluorodeoxyglucose PET Imaging Acquisition

FDG PET images were acquired using a Discovery 690 system (GE Healthcare). Three-dimensional FDG PET images were obtained after CT data were acquired for attenuation correction. In-plane and axial resolutions of the scanner were 4.9 mm and 5.6 mm full-width-half-maximum (FWHM), respectively. Images were reconstructed using a Gaussian filter (FWHM, 2 mm) and displayed in a 256 × 256 matrix. Emission images were reconstructed with OSEM using 24 subsets and 2 iterations.

### 2.5. ^18^F–Florbetaben PET Imaging Acquisition

β-amyloid PET images were obtained using Discovery 690, 710, or 690 Elite PET/CT scanners (GE Healthcare). Ninety minutes after the injection of 300 ± 30 MBq of ^18^F–Florbetaben, PET images were obtained for 20 min. Then, PET images were assessed visually by the 2 aforementioned nuclear medicine board-certified physicians to determine regional cortical uptakes in the frontal, lateral temporal, precuneus/posterior cingulate, and parietal regions. Patients were assigned as β-amyloid PET positive if increased uptake in any of the 4 brain regions was noted.

## 3. Results

### 3.1. Case 1 (PCA)

A 49-year-old woman presented with a 1.5-year history of visuospatial dysfunction to the memory disorder clinic at Asan Medical Center, Seoul, Korea. She kept losing her way and could no longer wear her clothes or perform numerical calculations. On neurologic examination, she showed partial Gerstmann syndrome (Rt.-Lt. disorientation, acalculia, and dysgraphia), Balint syndrome (ocular apraxia and simultagnosia), and severe dressing apraxia. In line with the clinical presentations, the neuropsychological test revealed parieto-occipital and frontal dysfunction. Detailed neuropsychological test results are described in Table 1. 

**Figure 1:**
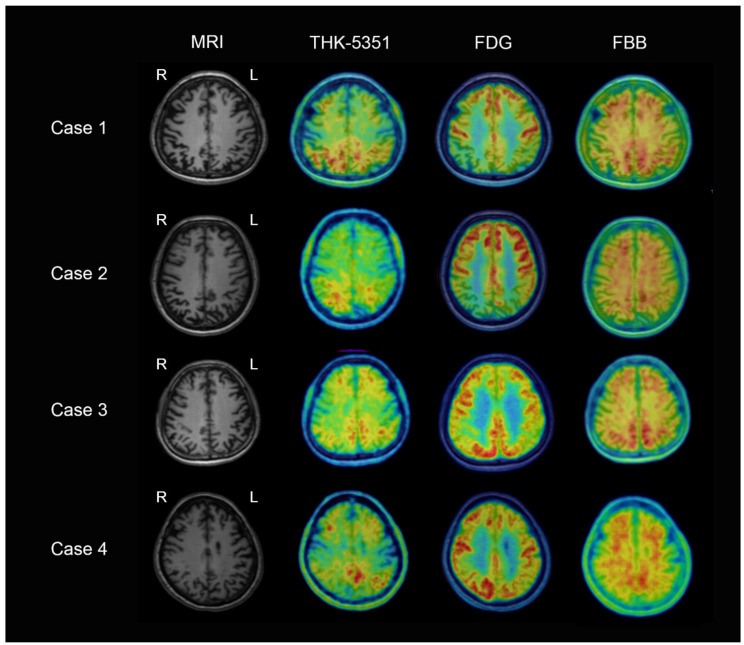
Axial structural magnetic resonance imaging (MRI) and three positron emission tomography (PET) images using ^18^F–THK–5351, ^18^F–Fluorodeoxyglucose, and ^18^F–Florbetaben in atypical Alzheimer’s disease cases. Cases 1 and 2: Posterior cortical atrophy patients demonstrating bilateral parieto-occipital cortical atrophy on structural MRI scan, parieto-occipital and frontal tau accumulation (posterior>frontal) on ^18^F–THK–5351 PET, glucose hypometabolism in both fronto-parieto-temporal association cortices, including the posterior cingulate cortex, on ^18^F–FDG PET, and increased amyloid uptake in diffuse cortices on ^18^F–FBB PET images. Cases 3 and 4: Logopenic variant primary progressive aphasia patients showing left temporo-parietal atrophy on structural MRI scan, temporo-parietal and frontal tau depositions (Left > Right) on ^18^F–THK–5351 PET, decreased glucose metabolism in bilateral temporo-parietal (Left > Right) and left frontal cortices on ^18^F–FDG PET, and widespread amyloid deposits on ^18^F–FBB PET images.

Cortical atrophy was observed in the bilateral parieto-occipital regions on structural MRI scan, to a greater extent in the right hemisphere. Tau retention was selectively restricted in the posterior brain regions, which corresponded to clinically affected areas. ^18^F–FDG–PET demonstrated hypometabolism in both fronto-parieto-temporal association cortices, including the posterior cingulate cortex. In contrast, the FBB PET image showed extensive and diffuse cortical ^18^F–FBB uptakes (Figure 1). 

### 3.2. Case 2 (PCA)

A 47-year-old woman visited the clinic with dysfunction in visuoperception. She could not read by line or recognize numbers or familiar objects. She presented all components of Gerstmann syndrome (Rt.-Lt. disorientation, acalculia, dysgraphia, and finger agnosia) and simultagnosia. Parieto-occipital and memory dysfunction were evident on the neuropsychological test.

### 3.3. Case 3 (lpvPPA)

A 58-year-old man presented with a 1.5-year history of progressive language disturbance. On language evaluation, he had severely impaired confrontational naming ability with decreased fluency and marked repetition errors. Because the neuropsychological test was performed 5 years after the symptom onset, it showed severely impaired language, memory, and frontal dysfunction, but relatively preserved visuospatial function (Table 1). 

Brain MRI demonstrated the asymmetric involvement of the fronto-temporo-parietal cortices in the left hemisphere, particularly in the left posterior temporal and parietal perisylvian regions. The ^18^F–THK5351 PET scan visualized asymmetric retentions that were prominent in the left temporal and parietal areas. FDG PET showed decreased glucose metabolism in the bilateral temporo-parietal, especially left, and left frontal cortices. On the contrary to the tau uptake, there were markedly increased ^18^F–FBB bindings throughout the association neocortices (Figure 1).

### 3.4. Case 4 (lpvPPA)

A 56-year-old woman visited the memory disorder clinic with progressive language disturbance. Language evaluation revealed decreased amount of speech with hesitation and impaired confrontational naming. Repetition of short phrases was also impaired. Global cerebral functions, including language, were impaired on the neuropsychological test (Table 1). 

There was asymmetric atrophy of fronto-temporo-parietal cortices in the left hemisphere, which was severe in the left posterior temporal and parietal peri-sylvian regions in the structural MRI scan, and corresponding glucose hypometabolism in the ^18^F–FDG PET. The ^18^F–THK–5351 PET scan showed temporo-parietal and frontal tau accumulation, more prominent in the left hemisphere. However, there was widespread amyloid distribution on FBB PET imaging (Figure 1).

## 4. Discussion

In this study of focal AD syndromes, we compared tau retentions, glucose metabolism, and amyloid deposition in PCA and lpvPPA using three different PET tracers, ^18^F–THK–5351, ^18^F–FDG, and ^18^F–FBB. Whereas the amyloid deposition was diffusely spread in the neocortex, glucose hypometabolism and increased tau uptake had focal distributions in the bilateral occipito-parietal regions in PCA and highly asymmetric left temporoparietal involvement in lpvPPA consistent with the clinical phenotypes. Based on the pathophysiology of the disease, it is plausible that the tau accumulation would precede the glucose hypometabolism. However, we could not conclude whether it happened in our image findings. Overall, tau accumulation was the mirror image of glucose hypometabolism, but it tended to be more restricted to the responsible area for clinical symptoms. This demonstrates that tau pathology represents clinical phenotypes better than amyloid pathology or glucose hypometabolism. 

This distinct pattern in PET imaging was consistent with those reported in previous studies using different amyloid and tau tracers. In two studies of atypical AD, including PCA and lpvPPA, an ^18^F–AV1451 tau PET scan showed closer association with the glucose hypometabolism and cognitive deficits than did amyloid deposition [14,15]. In another study using ^18^F–FDG and Pittsburgh compound B (PIB), there was syndrome-specific glucose hypometabolism in the patieto-occipital lobes in 13 PCA patients and asymmetric involvement of the left temporoparietal regions in 12 lpvPPA patients. Diffuse patterns of PIB uptakes across the cortex in both groups were noted with additional uptakes in the occipital cortex in the PCA group [16]. Also, there were stronger associations between tau and the clinical severity of dementia compared to the associations between amyloid deposition and the clinical symptoms [16]. Recent multimodal PET imaging studies with increased numbers of subjects diagnosed with PCA and lpvPPA have also suggested that a close relationship exists between tau accumulation and glucose hypometabolism, particularly in brain regions with more profound tau accumulation [17,18,19].

Our study provided a unique opportunity to compare three different modalities of PET and correlate their findings with clinical symptoms in patients with focal AD syndromes. Interestingly, the diffuse accumulation of Aβ overlaps with tau and glucose hypometabolism distribution in all cases. In these atypical AD cases, widespread patterns of Aβ accumulation were noted, which is similar to the Aβ accumulation patterns in typical AD. This suggests that although the role of Aβ is similar in both typical and atypical AD, the different topography of tau pathology may culminate into distinct clinical symptoms in line with other studies [14,15,17] However, the interaction between amyloid and tau and their influences on neurodegenerative processes are still unclear. Furthermore, substantial evidence has suggested that additional pathologies beyond Aβ and NFT are not uncommon in the brains of patients with AD, and that these pathologies also contribute substantially to the progression of AD [20,21,22]. For example, the prevalence of limbic-predominant age-related TDP-43 encephalopathy (LATE) or Lewy body pathology has been described to be as high as 50% in the brains of AD patients [20,21,22]. 

In line with efforts for developing and adapting new diagnostic tools to assess AD brain pathologies, guidelines for appropriate assessment of atypical AD patients should also be established for better diagnosis and management [23]. Clinically, detailed history taking to assess the earliest clinical features of patients should not be underestimated even in the era of high-tech imaging modalities. Efforts for the best clinical diagnosis will optimize the selection and interpretation of PET tracers for differential diagnosis as well as for assessing underlying pathologies in the brain.

This study is limited by caveats of the ^18^F–THK–5351 tracer described above [10]. We interpreted the THK–5351 uptakes as tauopathy in the brain. However, the increased uptake of THK–5351 is more likely to be indicative of neuroinflammation in the brain, potentially including the core of NFT pathology in the brain [11]. Future longitudinal studies with larger sample sizes and new tau tracers such as PI–2620 will further define clinical implications of the current findings as well as the causal relationship among image findings [24]. Future studies in typical AD patients with visuospatial dysfunctions but not fulfilling the diagnostic criteria of PCA or studies in typical AD patients with language deficits but not sufficient for the diagnostic criteria of lpvPPA will also provide further understandings about the hierarchical relationship between the PET findings that we noticed in focal AD syndromes.

## Figures and Tables

**Table 1:** Demographic and neuropsychological data.

	Case 1	Case 2	Case 3	Case 4
Age/Sex	49/F	47/F	58/M	56/F
Onset age	47	44	53	54
Disease duration (years)	1.5	3	5	2
Education (years)	9	16	14	8
Initial presentation	Losing directions, dressing apraxia	Visual agnosia	Word finding difficulty	Language disturbance
Clinical diagnosis	PCA	PCA	lpvPPA	lpvPPA
MMSE	21(<0.01%ile)	23 (<0.01%ile)	8 (<0.01%ile)	17 (<0.01%ile)
SNSB subdomain (percentile (z-score))				
Stroop CR	<0.01 (−5.89)	< 0.01 (−5.15)	N.A.	<0.01 (−5.34)
K-BNT	1.3 (−2.23)	<0.01 (−11.75)	<0.01 (−11.77)	<0.01 (−3.98)
DS-F	63.89 (0.36)	41.99 (−0.20)	0.17 (−2.93)	5.49 (−1.60)
DS-B	1.5 (−2.17)	9.40 (−1.32)	N.A.	<0.01 (−4.00)
SVLT-immediate	43.64 (−0.16)	0.08 (−3.17)	<0.01 (−5.91)	0.73 (−2.44)
SVLT-delayed	1.04 (−2.31)	1.07 (−2.30)	0.02 (−3.61)	0.30 (−2.75)
SVLT-recognition	24.39 (−0.69)	8.97 (−1.34)	21.94 (−0.77)	59.73 (0.25)
RCFT-immediate	0.4 (−2.65)	0.03 (−3.45)	0.44 (−2.62)	0.85 (−2.39)
RCFT-delayed	0.19 (−2.89)	0.02 (−3.60)	0.02 (−3.49)	1.66 (−2.13)
RCFT-recognition	0.01 (−3.70)	0.85 (−2.39)	<0.01 (−5.15)	11.43 (−1.20)
RCFT copy	<0.01 (−14.24)	<0.01 (−23.49)	39.76 (−0.26)	<0.01 (−7.16)
COWAT (Animal)	6.63 (−1.50)	12.85 (−1.13)	N.A.	0.59 (−2.52)
COWAT (Supermarket)	3.59 (−1.80)	13.40 (−1.11)	N.A.	1.16 (−2.27)
COWAT phonemic total	3.9 (−1.76)	40.82 (−0.23)	N.A.	N.A.

Abbreviations: PCA, posterior cortical atrophy; lpvPPA, logopenic variant primary progressive aphasia; CR, color reading; K-BNT, Korean version of the Boston naming test; DS-F, digit span-forward; DS-B, digit span-backward; SVLT, Seoul verbal learning test; RCFT, Rey complex figure test; COWAT, controlled oral word association test; N.A., not applicable.

## Data Availability

Data will be available upon request.
